# HI-FEVER: a Nextflow pipeline for the high-throughput discovery and annotation of endogenous viral elements

**DOI:** 10.1093/bioinformatics/btaf610

**Published:** 2025-11-08

**Authors:** Laura Muñoz-Baena, Emma F Harding, Jose Gabriel Nino Barreat, Cormac M Kinsella, Aris Katzourakis

**Affiliations:** Department of Biology, University of Oxford, Oxford, OX1 3EL, United Kingdom; Department of Biology, University of Oxford, Oxford, OX1 3EL, United Kingdom; Department of Biology, University of Oxford, Oxford, OX1 3EL, United Kingdom; Life Sciences Department, Natural History Museum, London, SW7 5BD, United Kingdom; Department of Biology, University of Oxford, Oxford, OX1 3EL, United Kingdom; Department of Bioinformatics and Genetics, Swedish Museum of Natural History, Stockholm, SE-104 05, Sweden; Department of Biology, University of Oxford, Oxford, OX1 3EL, United Kingdom

## Abstract

**Summary:**

Endogenous viral elements (EVEs) offer valuable insights into virus and host evolution, but their detection remains computationally and biologically challenging. We present HI-FEVER, a user-friendly Nextflow pipeline for the discovery of EVEs in eukaryotic host genomes. HI-FEVER is highly parallelizable and customizable, ensuring computational efficiency while allowing researchers to fine-tune parameters to their specific needs. Its output provides a comprehensive analysis of discovered EVEs, including detailed annotations which can provide evolutionary insights. HI-FEVER scales seamlessly to handle millions of viral protein queries across multiple host genomes on both laptops and high-performance computing nodes.

**Availability and implementation:**

The HI-FEVER source code is available on GitHub at https://github.com/PaleovirologyLab/hi-fever. Minimal reference databases, test datasets and benchmarking results are hosted on the Open Science Framework at https://osf.io/y357r. A detailed wiki is available at https://github.com/PaleovirologyLab/hi-fever/wiki, including usage instructions, parameter descriptions, and guidance on interpreting outputs. The pipeline includes a Pixi environment compatible with Conda and Apptainer containerization, and Docker images. HI-FEVER has been tested on Linux, Windows (via WSL2), and macOS (Intel and ARM64).

## 1 Introduction

Endogenous viral elements (EVEs) can arise when viruses integrate into host germline DNA and are passed to offspring. They are a unique resource for studying virus diversity, the evolution of host genomes, and the interactions between immunity and infection (Katzourakis and Gifford 2010, Hayward *et al.* 2015, Kawasaki *et al.* 2021, Moniruzzaman *et al.* 2022). In vertebrates, endogenous retroviruses are the most abundant EVEs due to the obligate integration of retroviruses during infection (Blanco-Melo *et al.* 2024, Katzourakis and Gifford 2010), whilst non-retroviral EVEs occur more sporadically and can be harder to detect due to their extensive diversity and lower abundance (Katzourakis and Gifford 2010, Nino Barreat and Katzourakis 2024). Eukaryotic lineages can have distinct EVE landscapes, for example plant and protist genomes contain giant virus EVEs, while insect genomes have a high prevalence of RNA viruses (Gilbert and Belliardo 2022, Bellas *et al.* 2023).

Despite their importance, EVE discovery and annotation is challenging—EVE sequences are often highly divergent (20%–40% AA identity) from their nearest known relatives, usually evolve neutrally with few highly conserved regions, and gene exchange between viruses and hosts can lead to complex histories of genes encoded by virus genomes (Irwin *et al.* 2022).

There is no standardized method for EVE detection in paleovirology. Most studies rely on a combination of similarity or motif searches, followed by manual curation and comparison against reference databases (Kawasaki *et al.* 2021, Blanco-Melo *et al.* 2024, Nino Barreat and Katzourakis 2024). Early efforts typically focused on single host genomes or narrow viral lineages due to limited data availability and computational resources (Katzourakis *et al.* 2007, Horie *et al.* 2010, Katzourakis and Gifford 2010). As genomic data from hosts and viruses continue to expand, researchers have developed several computational pipelines to support higher-throughput EVE discovery.

Pipelines like RetroTector (Sperber *et al.* 2009), RetroSnake (Kabiljo *et al.* 2022), EEfinder (Dias *et al.* 2024), and CauliFinder (Vassilieff *et al.* 2022) are tailored to specific viral families while more recent tools like detectEVE (Brait *et al.* 2025) and DIGS2.0 (Blanco-Melo *et al.* 2024) have flexible inputs for broader viral groups (S5). Pipelines for viral discovery from metagenomics data can also incidentally detect some EVE sequences (Aylward and Moniruzzaman 2021, Charon *et al.* 2022, 2024, Yang *et al.* 2024); however, they often fail to handle the unique characteristics and complexities of EVEs like sequence degradation and duplications. While existing pipelines address important aspects of EVE discovery, there remains a need for a tool that integrates speed, customizable search parameters, and comprehensive reciprocal validation to support scalable and flexible analysis across diverse host–virus systems.

## 2 Software overview

**Figure 1.**
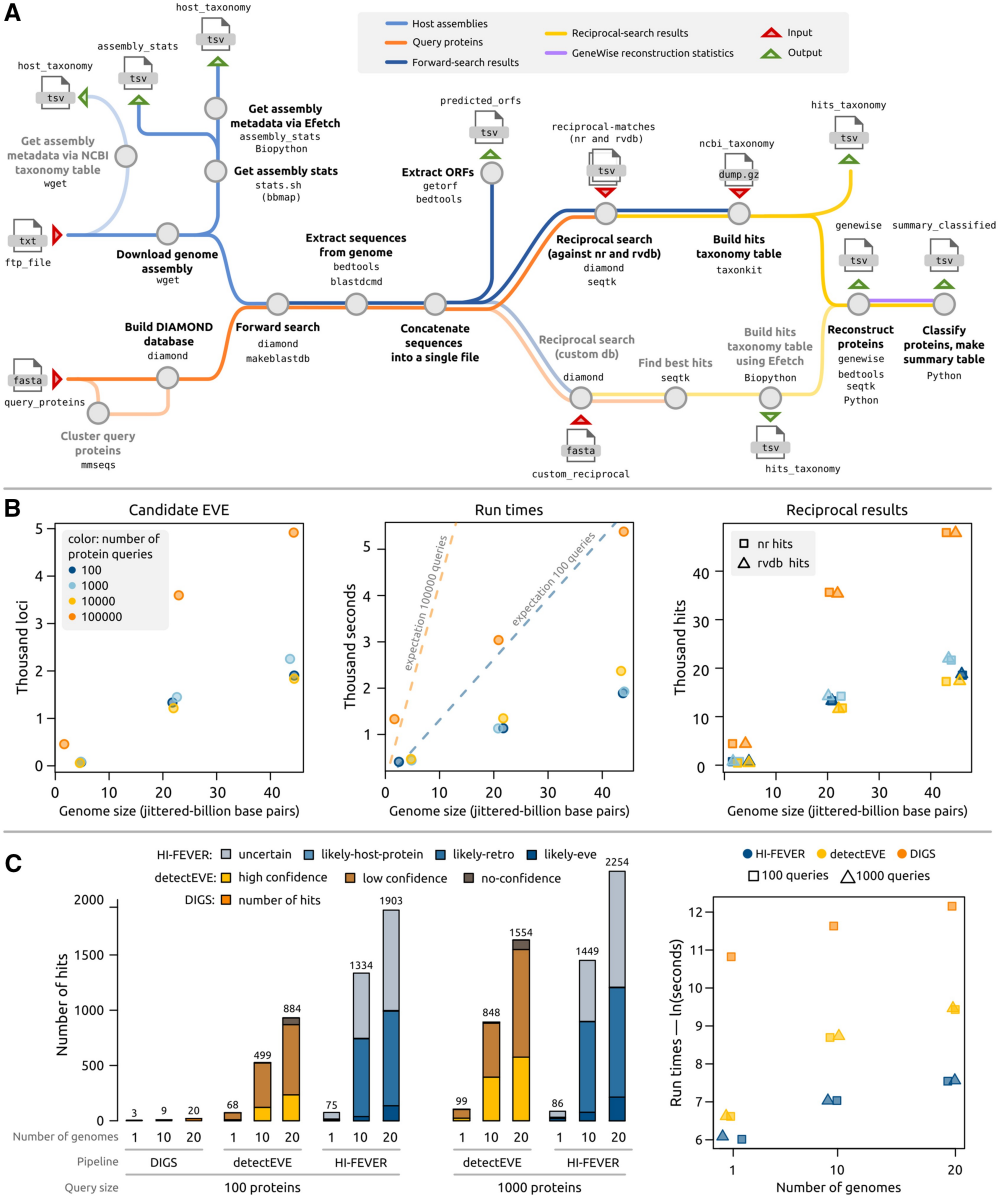
(A) HI-FEVER workflow. Given a set of query proteins and FTP links to host genome assemblies, HI-FEVER performs forward and reciprocal DIAMOND searches, reconstructs protein sequences from genomic regions identified as candidate EVEs, and classifies them based on reciprocal search results. Solid lines represent the default configuration; faint lines indicate alternative execution paths. (B) HI-FEVER performance. Number of candidate EVEs recovered and run times for query sets ranging from 100 to 100 000 proteins against 1, 10, and 20 host genomes. Dashed lines in the run time panel represent the expected linear increase as genomes are added. (C) Comparison with other EVE discovery pipelines. The same query sets used in the performance test were analyzed using DIGS and detectEVE across 1, 10, and 20 genomes, using a comparable setup. (Left) Number of candidate EVEs returned by each pipeline, grouped based on output annotation. (Right) Natural log-transformed run times (seconds) for each experiment. Note: the DIGS run for 1000 queries against one genome failed after 48 h due to a timeout in the SQL interface, so the experiments for this query size could not be compared. Plots created with R (4.1.2) (de Micheaux *et al.* 2013) and manually edited with Inkscape.

HI-FEVER (**Hi**gh-throughput Next**f**low **EVE R**ecovery) is a Nextflow pipeline (Di Tommaso *et al.* 2017) that integrates multiple tools to address the challenges of EVE detection (Fig. 1A). It retrieves protein and genome annotation data from NCBI using BioPython’s Efetch (Cock *et al.* 2009) and performs high-speed protein alignments with DIAMOND (Buchfink *et al.* 2021). For sequence extraction, it relies on BEDTools (Quinlan and Hall 2010) and Seqtk (Shen *et al.* 2016), and identifies open reading frames with getorf from EMBOSS (Rice *et al.* 2000). HI-FEVER also reconstructs candidate proteins using GeneWise (Birney *et al.* 2004), evaluating their length and intactness. Additionally, MMseqs (Hauser *et al.* 2016) is available as an optional step to cluster proteins and reduce redundancy in large input datasets.

Leveraging Nextflow’s parallelization capabilities, HI-FEVER runs several multi-core processes simultaneously, analyzes genomes independently, executes DIAMOND searches across multiple threads, and splits GeneWise predictions into parallel jobs. This approach ensures optimal resource utilization and minimizes overall runtime.

## 3 Implementation details

HI-FEVER consists of four adaptable and modular steps: (i) a similarity search to identify candidate EVEs, (ii) a reciprocal search against reference databases, (iii) protein reconstruction from genomic loci, and (iv) classification of candidates based on reciprocal search results.

Users can customise query sets, genome fragmentation size, ORF length thresholds, and the reference databases. This flexibility accommodates both exploratory and targeted analyses of specific viral groups. However, these settings can influence sensitivity and specificity and should be carefully selected. For example, retroviruses—due to their high copy number—can dominate search results if the genome is not sufficiently fragmented, potentially masking other EVEs. Using a vertebrate-only reciprocal database when analyzing fungal genomes could obscure relevant non-vertebrate hits. HI-FEVER is taxonomically agnostic by design, and users should customize inputs and parameters to their biological question to minimize bias and ensure interpretable results.

### 3.1 Forward search: similarity-based identification of candidate EVEs

The forward search in HI-FEVER identifies initial candidate EVEs by running a DIAMOND search (Buchfink *et al.* 2021) of the host genome assembly against a user-supplied set of viral proteins. All DIAMOND searches use an e-value threshold of $1\times 10^{-5}$ and report the top 10 hits ranked by bitscore.

To avoid bias toward highly similar or repetitive regions that can overshadow rare or low-identity EVEs, HI-FEVER splits each genome into fixed-size chunks (default: 50 000 bp). Each chunk is searched independently, returning a limited number of top hits (default: 1000). This strategy improves the detection of low-abundance or divergent EVEs by ensuring a more even representation of hits across large contigs.

### 3.2 Reciprocal-search: validating candidate EVEs

Due to the extensive gene exchange between viruses and hosts, some host genes have been captured by viruses, and vice versa. This evolutionary entanglement can lead to the identification of EVEs which are, in fact, host genes captured by virus genomes. HI-FEVER addresses this by performing a reciprocal DIAMOND search to better characterize candidate EVEs. Two modes are supported:

1. **Full reciprocal search (against nr and RVDB):** The default and most comprehensive option involves comparing candidate EVEs against both the Reference Viral Database (RVDB) (Bigot *et al.* 2019) and the NCBI non-redundant protein database (nr) (Celebi *et al.* 2023), clustered at 90% identity and 90% coverage. This provides complementary taxonomic information—i.e., RVDB offers high-confidence viral matches, while nr includes diverse non-viral proteins that help distinguish true viral integrations from host genes. For instance, candidate sequences with nr hits exclusively classified as “Viruses” likely represent genuine EVEs, while those with no RVDB hits but strong nr matches to eukaryotic proteins are likely to be of host origin. While highly informative, these databases are large (over 100 GB total), which may constrain users with limited computational resources. To address this, we provide a compact *minimal reciprocal* version derived from representative protein sequences across vertebrate genomes (clustered at 70% identity and 80% coverage), containing 4 423 506 proteins from nr and 65 321 from RVDB. The minimal nr database will introduce a vertebrate bias and we recommend using the complete versions of nr and RVDB when possible.
2. **Custom reciprocal search:** Users can provide their own curated protein datasets for the reciprocal search step to include viral or non-viral databases, or any combination relevant to the user’s research question.

### 3.3 Protein reconstruction

Endogenous viral elements accumulate missense and nonsense mutations over time that disrupt the original protein-coding sequences. HI-FEVER reconstructs the candidate EVE protein sequence using GeneWise (Birney *et al.* 2004), a widely used tool for predicting gene structure through comparison to protein sequence models. GeneWise reconstructs each EVE based on its closest related protein derived from the highest-scoring hit from either the forward or reciprocal DIAMOND searches. After reconstruction, HI-FEVER uses custom Python scripts to quantify the changes introduced—i.e., the number of stop codons, insertions or deletions, and frameshifts.

### 3.4 Classification of candidate EVEs

With information from the reciprocal-searches, HI-FEVER classifies candidate EVEs into one of six categories according to the following decision tree:

- **likely-retro:** If the annotation of hits to RVDB, at the family taxonomic level, contains the strings retro, or caulimo, **AND** if the names of the proteins in the RVDB or NR hits match the regular expression
gag|pol|env|reverse|pro[-]? pol|protease|pro|rt[-]? in.
- **likely-host-protein:** If there are no hits against the RVDB, **OR** if RVDB hits comprise less than 30% of the total hits to NR, **OR** if the protein names contain any of the strings ubiquitin, zinc, nynrin, or kinase.
- **bamford-related:** If any RVDB hit is annotated with a kingdom containing the string bamford. We introduced this category because these viruses frequently acquire host genes—making their sequences hard to classify without family-specific biological knowledge (Filée *et al.* 2008, Mönttinen *et al.* 2021).
- **likely-transposon**: If >80% of the RVDB hits are annotated as eukaryotic, **AND** the best RVDB hit title contains the strings pol, transpos, gag, or group specific antigen. This category accounts for loci with homology to retroviral proteins that originate from transposable elements—i.e., do not represent true viral integrations.
- **likely-eve**: For the remaining loci, if >60% of RVDB hits are viral and **none** of the families are annotated as baculo, poly, fabace, or allohe, **OR** if >50% of NR hits are classified as viral.
- **uncertain**: Assigned if none of the above conditions apply.

## 4 Usage

Users can clone the source code for HI-FEVER from https://github.com/PaleovirologyLab/hi-fever, install the basic dependencies via a Pixi or Conda environment, and run it from the command line as a Nextflow pipeline. It requires three mandatory inputs: (i) a FASTA file containing viral protein sequences; (ii) a text file with FTP links to NCBI host genome assemblies; (iii) and an email address for retrieving assembly and taxonomy metadata via Efetch—the NCBI tool for programmatically accessing their databases. Additional parameters can be specified in a custom configuration file (parameters.config).

### 4.1 Outputs

HI-FEVER produces detailed annotations of candidate EVEs as tabular and sequence data that can be integrated into relational databases for downstream analysis, including: (i) open reading frames (ORFs) with genomic coordinates and translated protein sequences, (ii) DIAMOND search results from the forward and reciprocal searches, (iii) reconstructed proteins with annotations for frameshifts, stop codons, and introns, and (iv) host assembly metadata and taxonomy of both host and protein hits. Additionally, HI-FEVER compiles the most relevant information into a summary table and FASTA files containing the peptide and cDNA sequences for each candidate EVE. These outputs support a range of downstream applications, including phylogenetic analysis, orthology inference, and detection of EVE copies within and across genomes.

Further details on output tables are available at https://github.com/PaleovirologyLab/hi-fever/wiki/Result-tables-explained

### 4.2 Test datasets

A folder with inputs for a sample run is available at https://osf.io/y357r/files. This folder includes: (i) a text file with FTP links to human genome assemblies, (ii) a query set of 100 viral protein sequences, (iii) minimal DIAMOND databases for reciprocal searches (nr and rvdb), and (iv) the “taxdump.tar.gz” file containing taxonomic information for the viral hits.

## 5 Benchmarking HI-FEVER

We benchmarked HI-FEVER with respect to speed, accuracy, sensitivity, and annotation richness, on a dedicated server with 48 Intel^®^ Xeon^®^ Gold 5220R CPUs (2.20 GHz), 250.4 GiB RAM, Ubuntu 20.04.6 LTS (64-bit), and a 2 TB NVMe SSD. All tests used the same hardware and software environment. We used three host datasets containing assemblies from 1, 10, and 20 vertebrate genomes (3.1, 21.2, and 44.3 Gb total size, respectively) and evaluated six viral query datasets:

1. hundred-prots-5fam: 100 proteins sampled evenly from five viral families: *Bornaviridae*, *Circoviridae*, *Filoviridae*, *Paramyxoviridae*, and *Parvoviridae*.
2. thousand-prots-5fam: 1000 proteins (200 per family) from the same five families.
3. ten-thousand-prots-5fam: 10 000 proteins (2000 per family).
4. hundred-thousand-prots-5fam: 100 000 proteins (20 000 per family).
5. thousand-prots-bamford: 1000 proteins from the viral kingdom *Bamfordvirae*, known for recurrent co-option of host genes (Filée *et al.* 2008, Mönttinen *et al.* 2021).
6. hundred-prots-plus-retro: 1200 proteins combining the hundred-prots-5fam dataset with 200 additional proteins from *Retroviridae*—a high-copy-number family that can obscure signals from other EVEs.

All the benchmarking queries, datasets, and results are available at https://osf.io/y357r/files.

### 5.1 Performance

HI-FEVER scaled efficiently across input sizes. The smallest run (hundred-prots-5fam) completed in 409 s. The subsequent runs were consistently faster than expected with additional genomes, indicating sublinear scaling between runtime and genome size (Fig. 1B).

Notably, the number of candidate EVEs did not scale linearly with the number of input queries. For example, when searching 20 genomes, 100 protein queries yielded 1903 candidate EVEs, whereas 100 000 queries yielded 4917—a modest increase given the 1000-fold rise in query size. This saturation likely reflects redundancy in large query sets. When we applied clustering to reduce query redundancy, HI-FEVER recovered more EVEs and completed runs faster, supporting the use of diverse, non-redundant query sets (S1).

### 5.2 Sensitivity analysis

We assessed HI-FEVER’s sensitivity by comparing its output to previously reported EVEs. It recovered all known *Bornaviridae*, *Circoviridae*, and *Parvoviridae* EVEs in the Tasmanian devil (*Sarcophilus harrisii*) genome (Dennis *et al.* 2019, Harding *et al.* 2021, Nino Barreat and Katzourakis 2024), as well as *Filoviridae* EVEs in the naked mole rat (*Heterocephalus glaber*) and American beaver (*Castor canadensis*) (Nino Barreat and Katzourakis 2024). We also detected *Circoviridae* EVEs in other genomes with previously reported integrations: dog (*Canis lupus*), frog (*Xenopus tropicalis*), pangolin (*Manis*), and sea otter (*Enhydra lutris*) (Liu *et al.* 2011, Dennis *et al.* 2019) (S2).

To assess the effects of query diversity on sensitivity and specificity, we used the thousand-prots-bamford and hundred-prots-plus-retro datasets. From the thousand-prots-bamford query set, HI-FEVER identified 13 654 candidate EVEs: 71.6% were classified as “likely-host-proteins,” 27.8% as “bamford-related,” and 0.3% as “likely-eves”—likely false positives due to the absence of known viral hallmark genes (S3).

From the hundred-prots-plus-retro dataset, HI-FEVER returned 75 113 candidate EVEs: 72.7% were classified as “likely-retro,” 12.1% as “likely-transposon,” and 13.7% as “uncertain.” The small fraction (0.16%) labeled as “likely-eve” included true positives from *Bornaviridae*, *Filoviridae*, *Parvoviridae*, and *Circoviridae*, showing that sensitivity was retained even when the search space was dominated by retroviral sequences (S4).

### 5.3 Comparison with other pipelines

We compared HI-FEVER to two other EVE-detection pipelines: DIGS (Database-Integrated Genome Screening) (Blanco-Melo *et al.* 2024) and detectEVE (Brait *et al.* 2025). All tools were run on the same datasets and hardware. To ensure consistency, we disabled detectEVE’s taxonomy filtering and masking, and we set defragment range to 1000 in DIGS to match HI-FEVER’s interval-merging behavior.

Among the three tools, HI-FEVER and detectEVE produced the most comparable outputs despite their differences in formats. HI-FEVER classifies hits into six categories: likely-retro, likely-host-protein, bamford-related, likely-transposon, likely-eve, or uncertain (see Implementation). In contrast, detectEVE applies a keyword-based scoring scheme and computes an EVE-score for each candidate locus. It then classifies each locus as high confidence, low confidence, or no-confidence; only loci from the first two classes are included in the final table. detectEVE writes results into a multi-FASTA file with coordinates in the headers, alongside a summary table with confidence scores and metadata.

In general, detectEVE and HI-FEVER reported similar numbers of candidate EVEs. When using the hundred-prots-5fam dataset across 1, 10, and 20 genomes, detectEVE classified 68, 497, and 827 candidate EVEs (plus 0, 2, and 57 no-confidence hits), while HI-FEVER identified 75, 1334, and 1903 candidates. DIGS returned only 3, 9, and 20, respectively. HI-FEVER completed the runs in 12, 18, and 32 min, and detectEVE took 12 min, 1 h 39 min , and 3 h 28 min. In contrast, DIGS required 13 h 56 min, 31 h 19 min, and 52 h 44 min (S6), reflecting the limitations imposed by the lack of workflow parallelization (Fig. 1C, S6).

All three pipelines found EVEs from *Bornaviridae*, *Parvoviridae*, and *Circoviridae*, but only HI-FEVER and detectEVE identified EVEs from *Filoviridae* and *Retroviridae*. The latter likely reflect cross-matches between filoviral glycoproteins and retroviral envelopes (Nino Barreat and Katzourakis 2024).

We also ran tests with the hundred-prots-plus-retro and thousand-prots-bamford query sets. For the query dataset including retroviruses detectEVE returned 9489, 91 584, and 174 794 candidate EVEs, validating 6325, 38 844, and 74 691 as high- or low-confidence. HI-FEVER returned 8766, 75 327, and 156 257 candidates but took slightly longer (Figure S7), likely due to its use of GeneWise for the amino acid reconstruction. In the thousand-prots-bamford tests, HI-FEVER finished faster and detected 182, 2501, and 4591 candidates, while detectEVE returned 115, 1423, and 2616 candidates, but validated none (Figure S8, S9).

The three benchmarking scenarios highlight the versatility of HI-FEVER across diverse taxonomical queries and under varying computational loads, including large numbers of viral queries and genomes. For both non-retroviral EVEs and NCLDVs, HI-FEVER was faster and recovered more candidate elements than detectEVE. It also provided additional context for ambiguous hits that may represent horizontal gene transfer events—in the NCLDV dataset, HI-FEVER classified these as either bamford-related or uncertain, whereas detectEVE excluded them from its final table due to failing validation as true EVEs. This reflects a key methodological difference—HI-FEVER annotates all hits whilst detectEVE only annotates hits beyond a confidence threshold.

When including retroviruses, HI-FEVER runtimes increased likely due to the computational demands of GeneWise-based protein reconstruction, and the total number of candidate EVEs was slightly lower than those found by detectEVE. However, after classification detectEVE reported fewer validated EVEs, while HI-FEVER identified most as either “likely-retro” or “likely-transposons.”

Overall, HI-FEVER is a versatile and accessible platform for endogenous viral element discovery, efficiently utilizing computational resources and scaling from laptops to high-performance clusters. Its classification framework and rich output annotations offer valuable, comprehensive information for large-scale EVE discovery and downstream analysis.

## Supplementary Material

btaf610_Supplementary_Data

## Data Availability

HI-FEVER is open source and available on GitHub at https://github.com/PaleovirologyLab/hi-fever. Minimal reference databases, test datasets and benchmarking results are hosted on the Open Science Framework at https://osf.io/y357r. A detailed wiki is available at https://github.com/PaleovirologyLab/hi-fever/wiki, including usage instructions, parameter descriptions, and guidance on interpreting outputs.
