# Supplementary material for: HI-FEVER: a Nextflow pipeline for the high-throughput discovery and annotation of endogenous viral elements
Source: Bioinformatics. 2025 Nov 8;41(12):btaf610. doi: 10.1093/bioinformatics/btaf610 (PMC12707981; doi:10.1093/bioinformatics/btaf610)
Supplement: btaf610_Supplementary_Data [file btaf610_supplementary_data.zip › supplementary_figures_S7_S8.pdf]

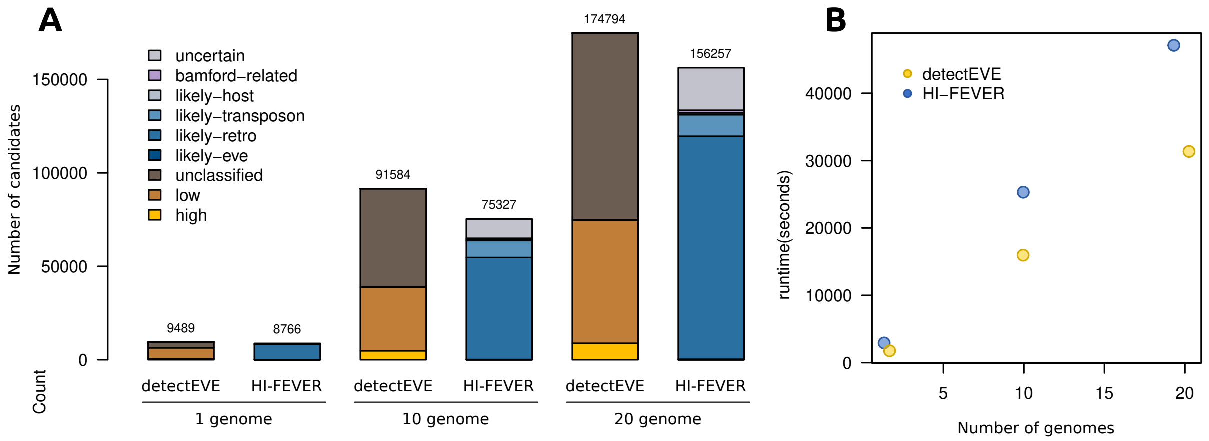

Figure S7 : **Benchmarking results with Retroviruses.** A viral protein query set including 200 proteins randomly sampled from each of the following families: *Parvoviridae*, *Bornaviridae*, *Filoviridae*, *Circoviridae*, *Paromyxoviridae* and *Retroviridae* (total 1200 proteins) was tested against 1, 10 and 20 vertebrate genomes using both HI-FEVER and detectEVE tools. **A)** Number and classification of candidate EVEs. **B)** Comparable runtime of EVE-detection tools.

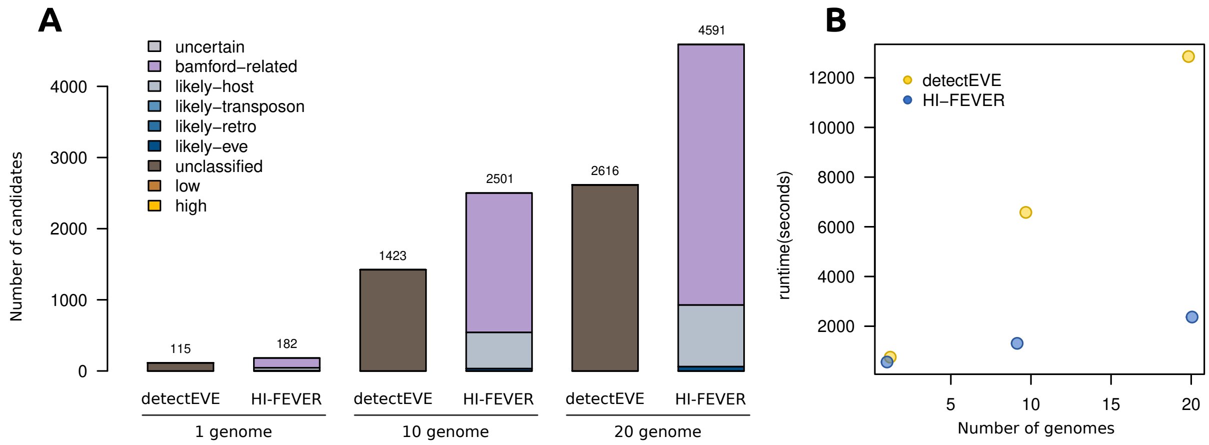

Figure S8 : **Benchmarking of *Bamfordvirae*-related elements.** A viral protein query set including 100 proteins randomly sampled from Bamfordvirae was tested against 1, 10 and 20 vertebrate genomes using both HI-FEVER and detectEVE tools. **A)** Number and classification of candidate EVEs. **B)** Comparable runtime of EVE-detection tools.
